# Chronic Lymphatic Leukemia Complicated with Priapism

**Published:** 1915-04

**Authors:** D. S. Hepburn

**Affiliations:** Buffalo. N. Y.; Pathologist of Buffalo Homoeopathic Hospital


### Chronic Lymphatic Leukemia Complicated With Priapism.

By D. S. HEPBURN, M. D., Buffalo, N. Y.

Pathologist of Buffalo Homoeopathic Hospital.

The average classification of the causes of priapism by most writers has been rather cumbersome. Those who are interested in this subject will find a very concise article by Frank Hinman in the "Annals of Surgery," December, 1914, in which he gives this classification:

Cases due to nervous causes.

Cases due to mechanic causes.

Mechanic cases subdivided into a. Thrombosis.
b. Haemorrhage and hematuria.
c. New growths of the penis.
d. Inflammatory swellings and edema of the penis.

27% of his series was associated with leukemia and gout. 70% had a duration of 20 to 60 days. Age most frequent between 20 and 50 years.

History of the patient E. M.; Age 52, occupation, policeman. Only previous illness muscular rheumatism, 5 years ago. Served on police force 30 years. Had been gaining weight and weighed as high as 286 in February, 1913. Three months ago began losing weight, and lost 60 pounds in three months. Markedly constipated. His family physician told him he was working too hard and gave him a tonic. Had attack of priapism 4 weeks ago. Appeared first in morning and lasted two hours. Appeared again February 17 upon waking; went to toilet and while trying to have stool had emission. Called Dr. Bowerman, police surgeon. Dr. Gardner saw him February 21, had marked priapism. Sent to Homoeopathic Hospital. Physical examination shows large spleen, coming down to level umbilicus; abdomen so fat impossible to say whether liver is enlarged. No enlargement of glands.

Hospital Report: Urinalysis: Quantity, (24 hrs.) 1000; color, yellow; specific gravity, 1025; reaction, acid; percentage of solids, 5.8; total solids, 58 grams; percentage of urea, 3.; total urea, 30 grams; percentage of uric acid, .176; total uric acid, 1.7 grams; chlorine, 3.2 grams; phosphoric acid, 1.3 grams; albumin, slight trace; sugar, absent.

Sediment: Casts, occasional hyaline; leucocytes, occasional; few disks; abundant amorphous urates.

On account of the principal symptoms complained of by the patient, viz.: priapism, a leukemic condition was thought of. The blood was accordingly examined and the results were as follows: Haemoglobin, 70; disks, 3,700,000; leucocytes, 1,014,000; small lymph, 95; large lymph, 3; neutrophiles, 2.

Showing a typical case of Chronic Lymphatic Leukemia. Because of leukemia being found the cause of the priapism was felt to be mechanical.

Operation, February 21st; corpus cavernosum opened, and about two ounces of thick gumous bloody clot removed.

The blood condition that this patient suffered from, viz.: Chronic Lymphatic Leukemia, is characterized by the following: The red cells show a moderate anemia and a few normoblasts may be present, but the changes are not so marked as in the acute stage. The important changes concern the lymphocytes and chiefly the small variety which are enormously increased. A comparison of the normal blood count with Chronic Lymphatic Leukemia may be of interest at this stage:

Normal Blood Shows: Color Index of 1; red cells, 5,000,000; total leucocytes, 7,000 to 10,000; large and small lymphocytes, 28 per cent.; polynuclears, 75 per cent.

Chronic Lymphatic Leukemia Shows: Color Index below 1; red cells, 2,800,000 average; total leucocytes, 180,000 (on 1st examination); large and small lymphocytes, 95 to 98 per cent.; polynuclears, 2 to 5 per cent.

Cabot cites a case in which he counted 3,500 white cells consecutively and which showed 99.6 per cent. lymphocytes.

The points of interest in this case were the absence of enlarged peripheral lymph glands. 44 out of 46 of Cabot's cases showed enlargement whereas our case showed none.

The second point was the larger number of leucocytes than is usually found in this condition. In Osler's series of 34 cases, 180,000 leucocytes per cubic millimeter was the average count, and only one case rose above a million cells.

Thirdly, the priapism present. Cabot says that priapism due to leukemic infiltration or thrombosis of the corpora cavernosa has been repeatedly mentioned in literature but, strangely enough, did not occur in any of the 89 cases of his series.

Fourthly, the increased amount of uric acid in the urine, .17 per cent. as compared to .05 per cent. in normal cases. In Osler's work, the following is said of this condition: "The most striking and important feature in the urine is the great excess of uric acid due to the breaking down of the nuclei from the death of the white cells. In no other disease is so great an increase of uric acid regularly found and it is an interesting fact that most of the symptoms often attributed to an excess of uric acid in the system are not found in leukemia." In our case the uric acid was increased .17 per cent. as compared to .05 per cent.

